# Personalized Reconstruction of Genital Defects in Complicated Wounds with Vertical Rectus Abdominis Myocutaneous Flaps including Urethral Neo-Orifice

**DOI:** 10.3390/jpm11111076

**Published:** 2021-10-24

**Authors:** Raymund E. Horch, Ingo Ludolph, Andreas Arkudas, Aijia Cai

**Affiliations:** Department of Plastic and Hand Surgery and Laboratory for Tissue Engineering and Regenerative Medicine, University Hospital Erlangen, Friedrich Alexander University Erlangen-Nuernberg FAU, 91054 Erlangen, Germany; ingo.ludolph@uk-erlangen.de (I.L.); andreas.arkudas@uk-erlangen.de (A.A.); aijia.cai@uk-erlangen.de (A.C.)

**Keywords:** perineal reconstruction, VRAM flap, neourethra, urethral reconstruction

## Abstract

Non-healing extensive wounds in the perineal region can lead to severe soft tissue infections and disastrous complications, which are not manageable with conservative measures. Specifically in recurrent or advanced pelvic malignancies, irradiation often leads to extensive scarring and wound breakdown, resulting in significant soft tissue defects during surgical tumor excision. Among several surgical options to reconstruct the perineum, the transpelvic vertical rectus abdominis myocutaneous (VRAM) flap has proven to be one of the most reliable methods. Specific modifications of this flap allow an individualized procedure depending on the patient’s needs. We modified this technique to include the urethral orifice into the skin paddle of VRAM flaps in three patients as a novel option to circumvent urinary diversion and maintain an acceptable quality of life.

## 1. Introduction

Perineal, genital and vulvar or scrotal defects often occur after infections or cancer treatment and are difficult to handle by conservative measures. As for the former, Fournier gangrene, a necrotizing fasciitis of the perineum and external genitals, is a life-threatening disease, which mainly affects male patients. Its main treatment includes aggressive debridement, resulting in extensive soft tissue defects in the perineal region [1]. As for the latter, irradiated relapsing vulva, anal or rectal cancer can lead to severe soft tissue infections and disastrous wounds that significantly impede the patient´s quality of life. In current modern oncological concepts of far advanced cases of pelvic malignancies, chemoradiotherapy is an established therapy. It may provide temporary symptomatic relief; however, it can deteriorate existing wounds [2,3,4,5,6,7]. Neoadjuvant radiotherapy can lead to wound breakdown, urinary and sexual problems, as well as postoperative bowel dysfunction [8]. Furthermore, exposition of the pelvic floor after pelvic exenteration often creates a risk of intestinal fistulas, and prior irradiation increases this risk [9].

The usefulness of a transpelvic vertical rectus abdominis myocutaneous (VRAM) flap has been extensively demonstrated as a method of choice to revascularize irradiated pelvic floor defects and to reconstruct the vagina [6,10,11,12,13,14]. From our experiences with more than 300 patients receiving VRAM flaps for pelvic, perineal and vaginal reconstruction, we previously published the reconstruction of postoncological perineal and vaginal defects in 142 female patients [12]. The more seldom extrapelvic route of VRAM flap transfer has been described in cases where laparotomy is not necessary for tumor resection [15]. In the case of perineal defects due to Fournier gangrene, the VRAM flap has only been used for extensive defects due to its bulkiness [16,17].

The majority of patients with pelvic malignancies who undergo exenteration need urinary diversion, e.g., in the form of an ileocolonic reservoir [18]. However, this often comes with complications such as ureteral strictures, pyelonephritis, difficulty in catheterization, or urinary stones [19]. Health-related quality of life is known to be negatively affected by a urostomy, leading to less participation in recreational activities and avoidance of social relationships [20].

We report on the extension of the surgical algorithm in reconstruction of complicated perineal wounds with VRAM flaps, utilizing an extrapelvic or transpelvic route combined with the creation of a neo-urethral orifice into the skin paddle of the VRAM flap in three patients. With this novel method, one can circumvent urinary diversion and maintain a better quality of life.

## 2. Patients and Methods

### 2.1. Technique of Surgery

The technique of harvesting of the VRAM flap and its translocation to the perineal area in an intrapelvic manner has been described in detail [10,21,22]. Briefly, a vertically oriented abdominal skin island is planned according to the (prospective) defect size in the perineal area and placed over the rectus muscle, preferably the right one. After the skin island is incised down to the anterior rectus sheath, the rectus muscle is raised from top to bottom after ligation of the superior epigastric vessels. During this procedure, the posterior rectus sheath is left intact and the skin island remains connected to the underlying muscle. The inferior epigastric vessels are visualized and dissected down to the external iliac vessels.

In the case of pelvic exenteration, a transpelvic translocation of the VRAM flap is used, and the attachment of the rectus abdominis muscle to the pubic bone is released, giving care to preserve the vascular pedicle by leaving the pyramidal component of the muscle insertion. Depending on the area to be reconstructed, the flap is then rotated at 180 degrees either into the pelvic cavity so that the skin paddle closes the defect with the proximal part facing the sacrum or it is transposed into the defect via a wide subcutaneous tunnel. By splitting a part of the skin paddle, a neovaginal orifice can be created if necessary.

When the urethra is maintained with a sufficient stump as in this series, a urethral orifice is created in the middle of the skin flap. The prospective location of the neourethra is marked on the skin paddle and the rectus muscle is carefully incised, preserving the epigastric vessels. The incision is continued through the skin paddle. A urinary catheter is pulled through the created orifice and placed into the remaining urethra. After the VRAM flap is sutured into the defect site, the bulky fat tissue of the skin island overlying the new orifice is carefully removed so that the urethral tissue can be sutured to the overlying skin with absorbable sutures.

**Figure 1:**
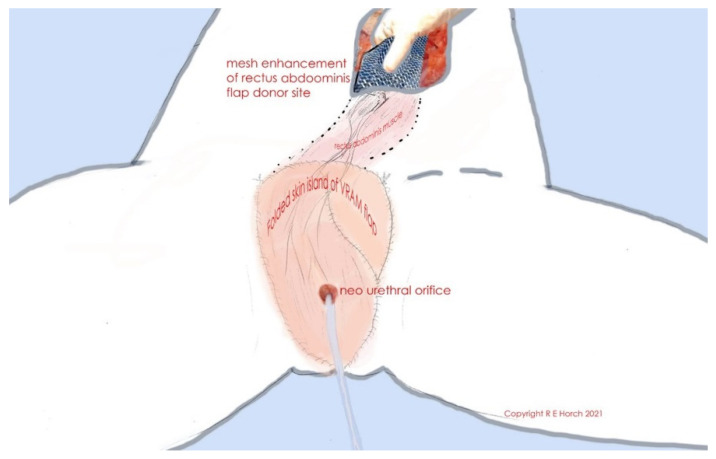
Schematic illustration of folded skin island of VRAM flap and neourethral orifice as well as of rectus abdominis muscle, tunneled subcutaneously. The flap donor site is closed with alloplastic mesh.

An extrapelvic route can be used in cases where there is no need to occlude any dead space in the pelvis as is presented in this series. In this case, attachment of the rectus abdominis muscle to the pubic bone is left intact and a subcutaneous tunnel is created between the rectus muscle and the tissue of the pubic region until the defect site is reached. The muscle is carefully pulled through the subcutaneous tunnel, giving care not to stress the vascular pedicle. The neourethra can be reconstructed in case of a sufficient remaining urethral stump, as described above (Figure 1).

In both cases, the rectus sheath is reconstructed with an alloplastic mesh and the abdominal skin can be directly closed [22]. If necessary, indocyanine green angiography can be used to confirm adequate flap perfusion at the end of the surgery [23,24].

### 2.2. Case 1

A 67-year-old female patient with recurrent melanoma of the vulva had undergone prior skinning vulvectomy, inguinal lymphadenectomy, radiotherapy, and chemotherapy. She further developed liver metastasis and chronic pain of the vulva, impeding sitting. A necrotic tumor, measuring 6 cm could be inspected in the vaginal introitus. An interdisciplinary surgical procedure was performed with the gynecologists, which included radical vulvectomy, bilateral colpectomy, resection of the distal urethral, which was also infiltrated by the tumor. The resulting defect was reconstructed with an extrapelvic VRAM flap with a 30 × 12 cm^2^ big skin paddle. Because intraoperative frozen sections of the resection margin of the distal urethra were tumor-free, a reconstruction of the remaining urethra was performed. For this purpose, a urinary catheter was tunneled through the middle of the VRAM flap, protecting the vascular pedicle, and inserted into the bladder through the remaining urethra. During the postoperative course, the VRAM flap healed without any complications, and the urinary catheter remained in the flap until the patient was discharged. A cystoscopy before discharge revealed no abnormalities. The patient received adjuvant chemotherapy in the Department of Dermatology and succumbed half a year later due to progressive multiple metastases, but expressed a comparatively good quality of life in terms of the defect reconstruction and the possibility to urinate through the neo-orifice.

### 2.3. Case 2

A 79-year-old female patient with recurrent vulva carcinoma had undergone multiple tumor excisions, lymphadenectomy, and radiotherapy. Another tumor recurrence eventually led to colpectomy, partial urethral resection, and brachytherapy. Half a year later, she was diagnosed with another recurrence, occupying the whole right labia. Furthermore, the rectum was infiltrated by the tumor. An interdisciplinary surgical procedure was performed with the gynecologists and the general surgeons, which involved a radical vulvectomy and excision of the rectum, respectively. Parts of the remaining urethra were also excised and analyzed via intraoperative frozen sections, which showed no signs of tumor infiltration. The resulting defect was reconstructed with a transpelvic VRAM flap with a 21 × 7 cm^2^ big skin paddle according to the defect size. A neourethra was created with the remaining parts of the urethra as described above. A urinary catheter was inserted through the neourethra in the VRAM flap and into the remaining urethra. The VRAM flap healed without any complications and the patient succumbed one month later due to the progressive tumor disease.

### 2.4. Case 3

**Figure 2:**
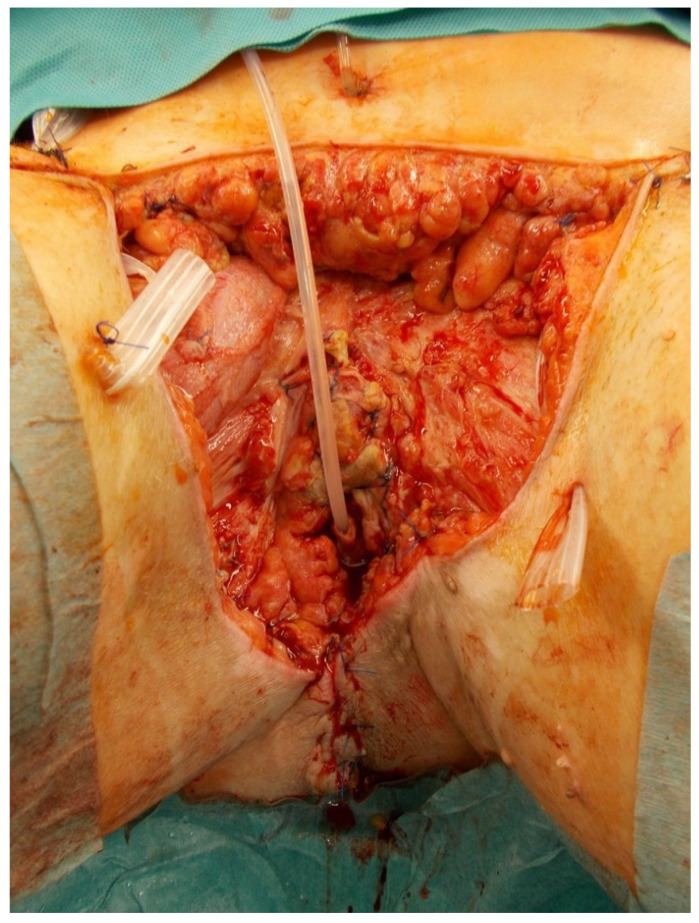
Extensive defect after radical debridement including penectomy for Fournier gangrene in a 68-year-old male patient. Urinary catheter is inserted into the remaining urethra.

A 68-year-old male patient with a history of dilated cardiomyopathy, atrial fibrillation, hypertension, diabetes, and obesity developed Fournier gangrene after left epididymitis, which led to radical debridement of the perineal region, including left orchiectomy and penectomy (Figure 2).

**Figure 3:**
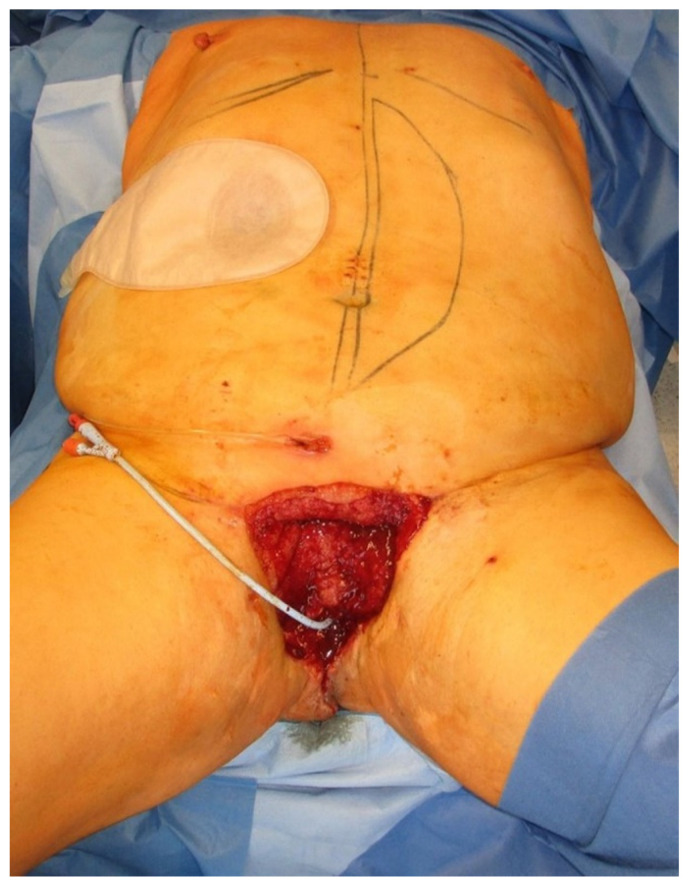
After adequate debridement, defect reconstruction was planned with an extrapelvic VRAM flap from the left abdomen. Skin paddle is marked.

He received a suprapubic cystostomy and a diverting colostomy. After the wound was conditioned via vacuum-assisted closure (Figure 3), reconstruction of the extensive wound was performed via an extrapelvic VRAM flap (Figure 1).

**Figure 4:**
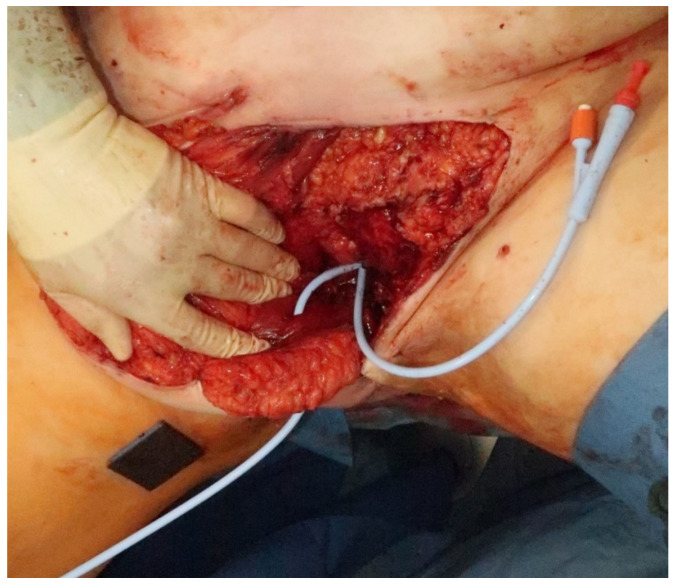
The VRAM flap was tunneled subcutaneously in an extrapelvic route to be placed into the perineal defect. A urinary catheter was tunneled through the middle of the VRAM flap and into the bladder through the remaining urethra. The old urinary catheter is still in place.

**Figure 5:**
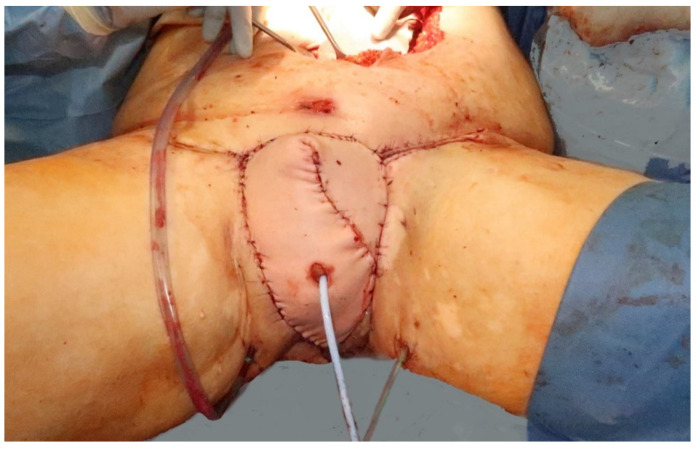
Immediate postoperative results after flap inset and reconstruction of the neourethra.

A urinary catheter was tunneled through the middle of the VRAM flap, protecting the vascular pedicle, and inserted into the bladder through the remaining proximal urethra, measuring 2 cm (Figure 4). Afterwards, the flap was sutured into the defect and showed adequate perfusion (Figure 5).

A few days later, the patient developed a hematoma under the flap, making surgical revision necessary. After hemostasis, the resulting wound was partially left open due to excessive swelling of the flap. Vacuum-assisted closure was applied and when swelling was reduced after several days, the defect was partially closed and partially reconstructed with a split-thickness skin graft. The patient developed renal insufficiency, which was managed by fluid resuscitation. A symptomatic pleural effusion was drained adequately. The further postoperative course was then uneventful and both the flap and the skin graft healed properly. The patient developed melanoma of his eye a few years later, leading to enucleation of his right eye. He paid regular visits to the urological department for changing his urinary catheter and was satisfied with the neo-orifice in the VRAM flap that he felt as an approvement compared to his intermediate previous situation.

## 3. Discussion

The VRAM flap has been a valuable tool for pelvic floor reconstruction in conjunction with oncological surgery of pelvic malignancies [6,10,12,21,22]. Gentileschi et al. have alternatively propagated the use of anterior lateral thigh (ALT) perforator flaps for reconstruction after vulvar cancer extirpative surgery due to a lower donor site morbidity [25]. There are certainly several reconstructive options to deal with defects in this context [21]. The VRAM flap is particularly suitable for cases of simultaneous reconstruction after tumor resection through an open laparotomy. In cases of secondary reconstruction, where a laparotomy is not indicated, alternative flaps can be used. However, there may be some limitations to harvesting a pedicled flap from the thigh when neoadjuvant extensive irradiation of the groin or thigh has been performed and lymphatic backflow in the extremity is compromised, resulting in donor site complication [26]. In the case of pelvic exenteration dead space, Gentileschi et al. harvested vastus lateralis muscle with the ALT flap [25]. Similar to standard bowel diversions through a rectus muscle, the vastus lateralis muscle may act as an additional buffer unlike a fasciocutaneous flap. Thus, we believe in the benefits of well-perfused muscle as can be found in a (transpelvic) VRAM flap to reconstruct irradiated perineal, vaginal, and/or gluteal regions. We were able to show an enormous improvement of both wound healing and quality of life in female patients with advanced or relapsing rectal, anal, or vaginal cancer. Patients did not report significant impairment in terms of sexual function as is commonly the case after abdominoperineal excision or low anterior excision and neoadjuvant radiotherapy as part of rectal cancer treatment [8,12]. Obviously, VRAM flap reconstruction leads to a lower complication rate after radical pelvic exenteration than in patients without a flap reconstruction, even though the extent of resection is usually larger and cancer disease is more advanced in those patients receiving radical pelvic exenteration. In addition, pelvic floor repair with a transpelvic VRAM flap reduces the number of perineal herniations when compared to primarily closed patients without flaps [27]. Modifications of the flap include splitting the distal portion longitudinally to produce “tongue” flaps to resurface vaginal and anal surfaces and creation of a neovaginal orifice through the central portion [15,28]. To our knowledge, creation of a neourethra by suturing the remaining stump to the surrounding tissue of the skin island has not been described as a modification of the VRAM flap so far.

Bregendahl et al. were able to show urinary dysfunction is also common in women after treatment for rectal cancer, especially after preoperative radiotherapy [8]. However, other malignancies such as vulvar cancer also necessitate (partial) urethral resection in the case of tumor infiltration, which might cause urinary tract dysfunction [29]. Reports on urethral reconstruction are scarce. Franchi et al. created a urethral neomeatus with vaginal mucosa in cases of partial urethrectomy [29]. However, in cases of radical pelvic exenteration, this option is not available. In male patients, the radial forearm free flap is the gold standard for reconstruction of the penis as well as the neourethra after penectomy or in terms of gender assigning surgery [30]. Phalloplasty with a radial forearm free flap has even been reported in a case after penectomy for Fournier gangrene. The reconstruction was successful even though the patient presented with comorbidities typical for those patients experiencing Fournier gangrene [31]. However, in the acute setting when the patient is in need of a safe and reliable reconstruction of an extensive defect, microsurgical procedures are not an option in those multimorbid patients. Fournier gangrene is a rapidly progressive necrotizing fasciitis of the genital and perineal tissues with a high mortality rate. Initial treatment includes radical surgical debridement of the affected tissues, broad spectrum antibiotics, and cardiopulmonary support [1,16,32]. After wounds have stabilized, reconstruction is needed, preferably with a technically simple and safe method. Loose wound approximation or split thickness skin grafts are only feasible in small or superficial wounds [1]. An extensive wound as described in our patient (Case 3) necessitates a flap coverage. Fasciocutaneous flaps as the pudendal thigh flap provide good cosmetic outcome and intact sensation [1]. For its bulkiness, the VRAM flap has rarely been used because it does not mimic the normal scrotum in appearance [17]. However, in defects as presented in our case where a penectomy was necessary, the VRAM flap is a safe and reliable option to reconstruct the whole defect area in the morbid patient. The VRAM flap is traditionally delivered in a transpelvic manner, using an intraperitoneal route. In cases where laparotomy is not used for tumor resection, an extrapelvic route is preferred [15]. This is also the case for tumors limited to the anterior part of the pelvis as described in our first case of recurrent melanoma of the vulva without rectal infiltration or in our third case of extensive genital and perineal defects after radical debridement, including penectomy due to Fournier’s gangrene.

The limitations of this study include the short follow-up period of our first two cases due to the palliative situation. The first patient succumbed to her metastasized end-stage melanoma disease approximately 6 months after she was discharged from the gynecological department. We do not know if voiding without the urinary catheter was possible, but 2 months after the surgery, a cystoscopy could be performed easily through the neourethra. Cystoscopic findings and histological specimens of the remaining urethra did not reveal any pathologies. As described previously, long term observations of vaginal reconstructions with the VRAM flap showed that although the abdominal skin of the flap island is not primarily accommodated to the moist milieu of the vagina, the skin island seems to adapt to the new surrounding conditions even when the whole vagina was reconstructed with two flaps [33]. All in all, our study population was characterized by high morbidity and we were not able to quantify health-related quality of life with validated scores. However, all patients reported a subjective improvement of quality of life at follow-up visits, which were short for the majority of patients. The only alive patient with a potentially longer follow-up had multiple comorbidities typical for those suffering from Fournier gangrene and was diagnosed with melanoma a few years later. Documentations from his latest visits to the Department of Urology suggest regular changes of his urinary catheter. Removal of the catheter was not desired by the patient probably due to comfort reasons and because voiding without a penis might have imposed a great psychological challenge to the patient. A phalloplasty has not been desired by the patient so far. To evaluate the benefits of this modified technique of perineal and urethral VRAM flap reconstruction, a long-term follow-up of a set of younger and healthier patients would be necessary. However, Nigriny et al. reported on a case of perineal reconstruction with an extrapelvic VRAM flap and creation of a neovaginal and urethral orifice similar to our technique in a 54-year-old woman with anal squamous cell carcinoma. After a follow-up of 38 months, she maintained urinary continence. This case supports the viability of our technique [15].

When weighed against the gain in quality of life, the VRAM flap reconstruction is a readily available and safe tool to optimize the outcome even in palliative and multimorbid cases. All patients in this series described a subjective significant improvement in their quality of life by this repair technique. Taking the negative impact of a urostomy into account, one should always consider the reconstruction of a neourethra if possible in conjunction with a perineal VRAM flap reconstruction [20].

## 4. Conclusions

The VRAM flap is not only a safe and reliable reconstructive option for perineal defects following abdominoperineal excision—with or without vaginal wall resection—but also for extensive perineal defects after Fournier gangrene. In the latter case, an extrapelvic route is preferred. Modifications of the VRAM flap and further personalization of the surgical approach allow for a neourethral reconstruction in the case of available remaining urethral tissue. Whenever possible, this technique should be applied to ensure that future urinary continence may be maintained. This can add enormous improvements to the quality of life of those patients.

## Data Availability

The data presented in the study are available on reasonable request from the corresponding author. The data are not publicly available due to ethical reasons.
